# ICTV Virus Taxonomy Profile: Hantaviridae 2024

**DOI:** 10.1099/jgv.0.001975

**Published:** 2024-04-08

**Authors:** Steven B. Bradfute, Charles H. Calisher, Boris Klempa, Jonas Klingström, Jens H. Kuhn, Lies Laenen, Nicole D. Tischler, Piet Maes

**Affiliations:** 1 University of New Mexico Health Sciences Center, Albuquerque, New Mexico, USA; 2 Colorado State University, Fort Collins, Colorado, USA; 3 Biomedical Research Center, Slovak Academy of Sciences, Bratislava, Slovakia; 4 Linköping University, Linköping, Sweden; 5 Integrated Research Facility at Fort Detrick, Frederick, Maryland, USA; 6 University Hospitals Leuven, Leuven, Belgium; 7 Centro Ciencia & Vida, Fundación Ciencia & Vida, Santiago, Chile; 8 Universidad San Sebastián, Santiago, Chile; 9 Zoonotic Infectious Diseases Unit, Rega Institute, KU Leuven, Leuven, Belgium

**Keywords:** ICTV Report, *Hantaviridae*, hantavirus, orthohantavirus, taxonomy

## Abstract

*Hantaviridae* is a family for negative-sense RNA viruses with genomes of about 10.5–14.6 kb. These viruses are maintained in and/or transmitted by fish, reptiles, and mammals. Several orthohantaviruses can infect humans, causing mild, severe, and sometimes-fatal diseases. Hantavirids produce enveloped virions containing three single-stranded RNA segments with open reading frames that encode a nucleoprotein (N), a glycoprotein precursor (GPC), and a large (L) protein containing an RNA-directed RNA polymerase (RdRP) domain. This is a summary of the International Committee on Taxonomy of Viruses (ICTV) Report on the family *Hantaviridae,* which is available at ictv.global/report/hantaviridae.

## Virion

**Fig. 1.**
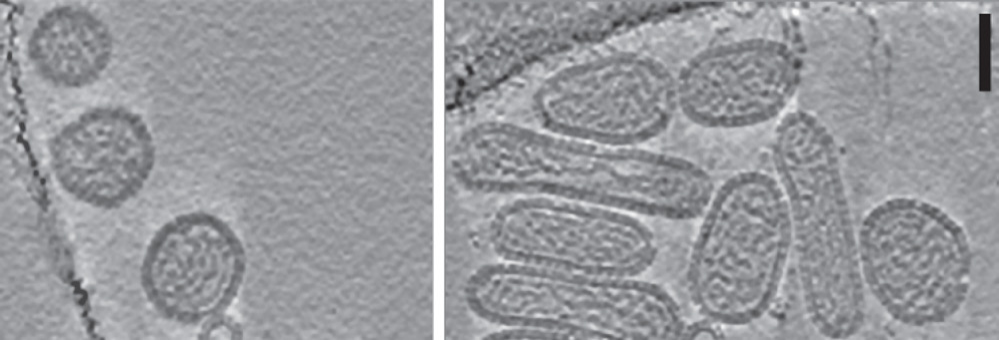
Electron micrograph of (mammalian) Tula virus particles, adapted with permission from [6]. Scale bar 50 nm.

Hantavirids produce virions that are pleomorphic in shape and 80–160 nm in diameter, with lipid envelopes (Table 1 and Fig. 1). Individual virions have a square lattice of tetrameric glycoprotein spikes composed of G_N_-G_C_ heterodimers derived from GPC. Isolated ribonucleoprotein (RNP) complexes are composed of individual segments of genomic RNA encapsidated by N and associated with L protein [1].

**Table 1.** Characteristics of members of the family *Hantaviridae*

Example	Hantaan virus (S: M14626; M: M14627; L: X55901), species *Orthohantavirus hantanense*, genus *Orthohantavirus*
Virion	Enveloped, pleomorphic virions 80–160 nm in diameter with heterodimeric surface spikes
Genome	Three single-stranded RNA molecules (segments): small (S: 1.0–3.0 kb), medium (M: 3.4–4.8 kb), and large (L: 5.3–6.8 kb)
Replication	Ribonucleoprotein (RNP) complexes contain antigenomic RNAs that serve as coding templates for the synthesis of genomic RNAs
Translation	From capped and non-polyadenylated mRNAs; the 5′-cap structure is obtained via cap-snatching from cellular mRNAs
Host range	Fish (e.g. actinoviruses, agnathoviruses), mammals (e.g. loanviruses, mobatviruses, orthohantaviruses, thottimviruses) and reptiles (e.g. reptilloviruses)
Taxonomy	Realm *Riboviria,* kingdom *Orthornavirae,* phylum *Negarnaviricota*, class *Ellioviricetes*, order *Bunyavirales*: the family includes >3 subfamilies, >6 genera, and >50 species

## Genome

**Fig. 2.**
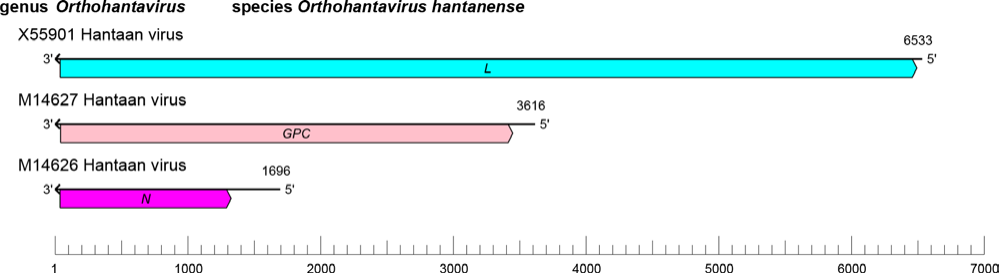
Genome organisation of Hantaan virus. *GPC*, glycoprotein precursor gene; *L*, large (protein) gene; *N*, nucleoprotein gene.

Hantavirids have tri-segmented, negative-sense RNA genomes (small [S], medium [M], and large [L] segments) (Fig. 2). These RNAs encode, respectively, in the virus-complementary sense, N, GPC, and L protein containing RdRP, helicase, and endonuclease domains. The S segment of some hantavirids encodes a nonstructural protein (NSs) [2].

## Replication

Hantavirid infection starts with virion attachment, mediated by G_N_ and G_C_. Protocadherin-1 is a clade-specific receptor of American orthohantaviruses required for entry into endothelial cells and alternative receptors may be used in other cell types. After uptake into the endosome, G_C_ drives membrane fusion with the host cell resulting in early or late endosomal release of the virion RNP complex into the cytoplasm in a pH-dependent manner [2]. During primary transcription, L generates uncapped antigenomic RNA molecules that are then capped using host cell-derived capped primers (cap snatching). L and S segment-transcribed mRNAs are translated by free ribosomes. M segment-transcribed mRNA is translated by membrane-bound ribosomes, with the expressed GPC cleaved likely by cellular proteases to yield G_N_ and G_C_. The synthesis of the antigenomic RNA by L protein serves as a template for genomic RNA replication. Secondary transcription amplifies the synthesis of mRNAs and genome replication [3]. During biogenesis, G_N_ and G_C_ traffic to the Golgi or ER-Golgi intermediate apparatus and upon interaction of their cytoplasmic tails with the viral ribonucleocapsids new virions bud from cellular membranes for their subsequent release; specific assembly sites remain to be established [3].

## Pathogenicity

Orthohantaviruses tend to cause chronic, largely asymptomatic infection in their host reservoirs. Human infection can occur after inhalation of aerosolized excreta of infected mammalian hosts. Two main syndromes have been identified: hantavirus pulmonary syndrome (HPS), caused by orthohantaviruses endemic in the Americas, and haemorrhagic fever with renal syndrome (HFRS), caused by orthohantaviruses originating in Europe and Asia [4].

## Taxonomy

Current taxonomy: https://ictv.global/taxonomy. The family *Hantaviridae* is included in the negarnaviricot order *Bunyavirales*. Within this order, hantavirids are most closely related to crulivirids, fimovirids, peribunyavirids, phasmavirids, tospovirids, and tulasvirids [5]. Like most other bunyavirals, hantavirids (i) have multi-segmented, negative-sense RNA genomes; (ii) encode proteins with high sequence identity; (iii) have five conserved motifs (A–E) in their RdRP domain; and (iv) produce enveloped virions.

## Resources

Full ICTV Report on the family *Hantaviridae*: www.ictv.global/report/hantaviridae.

## Abbreviations

G_C_: C-terminal glycoprotein
G_N_: N-terminal glycoprotein
GPC: glycoprotein precursor
HFRS: hemorrhagic fever with renal syndrome
HPS: hantavirus pulmonary syndrome
L: large protein
N: nucleoprotein
RNP: ribonucleoprotein
